# P-211. Impact of Disparities in Access to Antibiotics on Inferring Burdens of Difficult-to-Treat Antibiotic Resistance: A Real-world Data Analysis and Simulation Study

**DOI:** 10.1093/ofid/ofaf695.433

**Published:** 2026-01-11

**Authors:** Morgan Walker, Sarah Warner, Christina Yek, Sadia Sarzynski, Sameer S Kadri

**Affiliations:** National Institutes of Health, Bethesda, Maryland; NIH - Critical Care Medicine Department, Bethesda, MD; National Institute of Allergy and Infectious Diseases, Bethesda, Maryland; Critical Care Medicine, National Institutes of Health Clinical Center, Bethesda, Maryland; National Institutes of Health Clinical Center, Bethesda, MD

## Abstract

**Background:**

Global disparities in antibiotic access complicate inferences of antibiotic resistance (AMR) burdens. Difficult-to-treat resistance (DTR), or resistance to all highly safe and effective antibiotics, has clinical relevance and prognostic utility, but assumes these antibiotics are accessible. We propose a “DTR index” which for a pathogen is defined as the percent isolates with no accessible safe and effective antibiotic options in a region over a denominator of total isolates with available antibiotic susceptibility data. We tested how a DTR index changed over time with emergence of newer antibiotics in a real-world cohort of US hospital and across regions with simulated degrees of antibiotic access.

**Figure:**
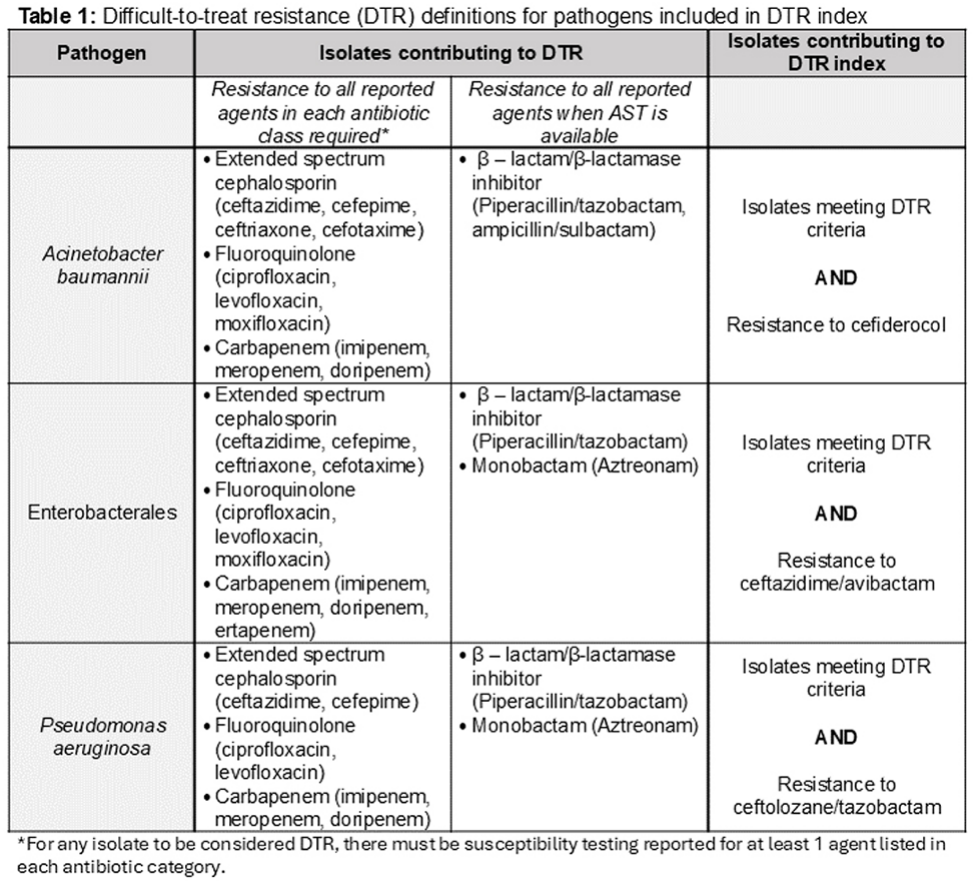

**Figure 1:**
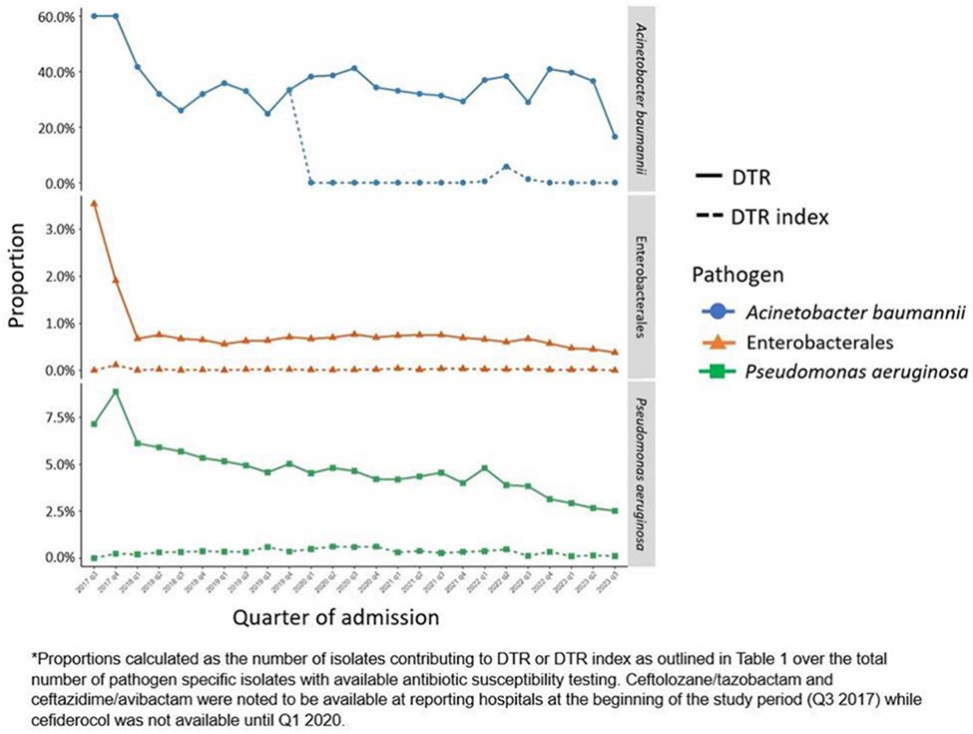
Trends in DTR proportion and DTR index following the introduction of new antibiotics*

**Methods:**

Clinical cultures with Enterobacterales, *P. aeruginosa*, or *A. baumannii* and susceptibility testing to ≥ 1 carbapenem, extended-spectrum cephalosporin, and fluoroquinolone were identified from 339 US hospitals in the PINC-AI healthcare database between 2017-2023. Determinants of the DTR index for each pathogen are provided in Table 1. Pathogen-specific trends in DTR proportion and DTR index were plotted by quarter with introduction of ceftazidime/avibactam, ceftolozane/tazobactam, and cefiderocol. Using microbiologic data in our US derived cohort, we developed 3 hypothetical scenarios to determine changes in the DTR index for Enterobacterales with decreasing antibiotic accessibility.

**Figure 2:**
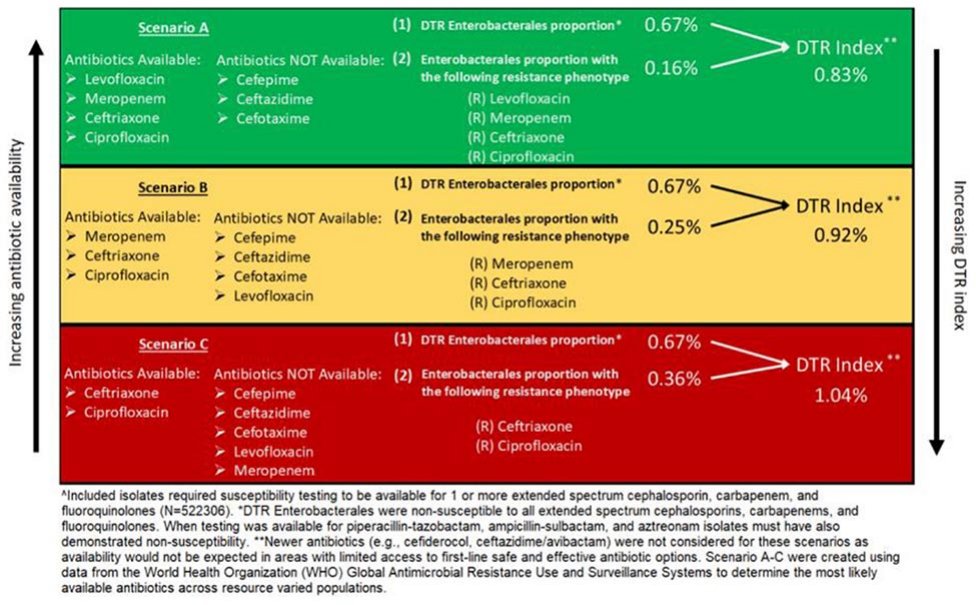
Three hypothetical scenarios for changing Enterobacterales DTR index based on accessible antibiotics^

**Results:**

The DTR index for Enterobacterales and *P. aeruginosa* remained substantially lower than the estimated DTR proportion between 2017-2023 given the availability of ceftolozane/tazobactam and ceftazidime/avibactam. In contrast, the DTR index for *A. baumannii* which assumes the value of the estimated DTR proportion until cefiderocol became available at reporting hospitals in 2020 and the DTR index decreases from 35% to nearly 0% (Figure 1). We also demonstrated an increase in the Enterobacterales DTR index in 3 hypothetical scenarios with varying levels of antibiotic accessibility (Figure 2).

**Conclusion:**

The DTR index offers a practical solution to gauge the effect of new antibiotics and the effect of regional differences in antibiotic access on AMR burdens. Its incorporation might help quantify efforts to improve global antibiotic access.

**Disclosures:**

All Authors: No reported disclosures

